# Gaussian graphical models for phenotypes using pedigree data and exploratory analysis using networks with genetic and nongenetic factors based on Genetic Analysis Workshop 18 data

**DOI:** 10.1186/1753-6561-8-S1-S99

**Published:** 2014-06-17

**Authors:** Rajesh Talluri, Sanjay Shete

**Affiliations:** 1 Department of Biostatistics, The University of Texas MD Anderson Cancer Center, Houston, TX, USA; 2 Department of Epidemiology, The University of Texas MD Anderson Cancer Center, Houston, TX, USA

## Abstract

Graphical models are increasingly used in genetic analyses to take into account the complex relationships between genetic and nongenetic factors influencing the phenotypes. We propose a model for determining the network structure of quantitative traits while accounting for the correlated nature of the family-based samples using the kinship coefficient. The Gaussian graphical model of age, systolic blood pressure, diastolic blood pressure, hypertension, blood pressure medication use, and smoking status was derived for three time points using real data. We also explored binary sparse graphical models of single-nucleotide polymorphisms (SNPs), covariates, and quantitative traits for exploratory analysis of the data. We validated the applicability of this method by producing a network graph using 20 causal variants, 21 noncausal variants, and 6 binary and quantitative phenotypes using the simulated data. To improve the model's ability to identify associations between the causal variants and the phenotypes, we intend to conduct follow-up studies investigating how to use the relationships between SNPs and between SNPs and phenotypes when analyzing genome wide association data with multiple phenotypes.

## Background

Graphical models are popular methods for exploratory data analysis [1]. Understanding the network structure of various genetic and nongenetic factors affecting phenotypes is gaining importance because more methods are developed that are capable of using such information. Analyzing high-dimensional data is a challenging task. Subset selection is a useful tool to remove noise in the data. LASSO-based methods [2]are very useful for reliable analysis of high-dimensional data. Here, we propose a model for determining the network structure of quantitative traits while accounting for the correlated nature of the family-based samples using the kinship coefficient.

## Methods

### Gaussian graphical models for quantitative traits in pedigrees

Suppose *Y *is a n×p data matrix containing *n *individuals and *p *quantitative traits (e.g., systolic blood pressure [SBP] and diastolic blood pressure [DBP]). The individuals are correlated because they were sampled from pedigrees. The correlation among the samples attributed to shared genetics and environment can be modeled using the kinship coefficient [3]. The kinship coefficient determines the genetic similarity or relatedness between 2 individuals within a pedigree. We intend to estimate the dependence structure between the traits while accounting for the correlation in the samples.

The data can be modeled using matrix normal distribution [4], which models the dependency structures; the dependency between the samples; and the dependency between the traits, $Y_{n\times p}\sim Matrix\;Normal\left(0_{n\times p},{\text{Ω}}_{p\times p}^{-1},R_{n\times n}\right),$ where $0_{n\times p}$ is the standardized mean, *Ω *is the inverse of the covariance of the *p *quantitative traits, and *R *is the covariance matrix of the *n *related samples. Because the individuals from different pedigrees are independent and the correlation exists only within a pedigree, *R *is a block diagonal matrix, with each block corresponding to a pedigree. Within each pedigree, the correlation coefficients between the samples are specified as twice the value of the kinship coefficient. Using reported heritability values of SBP and DBP and using phenotypic correlation between monozygotic twins [5], we estimated the shared environmental component to be approximately 0.1. Therefore, a constant c=0.1 was added to the correlation of samples within a pedigree to account for the shared environmental factors. The likelihood for the model is:$Likelihood\propto \text{det}{\left(\text{Ω}\right)}^{\frac{n}{2}}\text{det}{\left(R\right)}^{\frac{p}{2}}\text{exp}\left[-\frac{1}{2}\text{trace}\left(\text{Ω}{\text{Y}}^{\text{T}}{\text{R}}^{-1}\text{Y}\right)\right]$. The log likelihood can be written as $\text{log}\text{\_}likelihood\propto \frac{n}{2}logdet\left(\text{Ω}\right)-\frac{1}{2}trace\left(\text{Ω}Y^{T}R^{-1}Y\right)$.

Many methods are available to optimize *Ω *over the model's likelihood function. But to identify the conditional independence structure of the *p *traits, we have to find traits that are conditionally independent given all other traits. This information is contained in the inverse of the correlation matrix *Ω *between the traits. If ${\text{Ω}}_{i,j}=0$, trait *i *and trait *j *are conditionally independent, given all other traits. To enforce sparsity in the estimation of Ω, the LASSO penalty can be imposed on the likelihood. The resulting penalized log likelihood is $\text{log}\text{\_}likelihood\propto \frac{n}{2}logdet\left(\text{Ω}\right)-\frac{1}{2}trace\left(\text{Ω}Y^{T}R^{-1}Y\right)-\rho ∥\text{Ω}∥$, where $\;∥\text{Ω}∥$ is the L1 norm of Ω. The LASSO penalty estimates the sparse graphical model [6] of dependence between the quantitative traits by forcing the value of nonsignificant elements in the inverse of the covariance matrix to zero. At each time point, the precision matrix between the phenotypes was estimated using graphical lasso, penalization on the graphical model likelihood using the R package "glasso" [6]. Because the correlation among individuals within a family is not accounted for in the standard glasso, we modified this package to account for such relationships using the kinship coefficient. The kinship coefficient for individuals within the pedigrees was estimated using the SimWalk2 program [7].

### Sparse graphical models for binary and quantitative traits

The proposed model is not suitable for exploring the association between single-nucleotide polymorphisms (SNPs) and quantitative traits such as SBP and DBP. In the case of continuous traits, conditional independence between nodes is directly estimated using the partial correlation coefficients, which are related to the inverse of the correlation matrix. This interpretation of conditional independence is not possible, however, when the traits are discrete. The assumptions required for Gaussian graphical models are not satisfied for discrete phenotypes such as hypertension and smoking status or for SNP genotypes. Some of the popular approaches for estimating graphical networks of discrete variables are based on the LASSO regression [6,8].

Assume *Y *is a n×pdata matrix containing *n *individuals and *p *quantitative and discrete traits. The *p *variables, or nodes, of the network contain SNPs, quantitative phenotypes such as age, SBP and DBP, and binary phenotypes such as hypertension, blood pressure medication, and smoking status. Each of the variables (e.g., SBP, DBP, hypertension, age, SNPs) is considered as a response and is regressed on all of the other variables, which are considered as predictors. The predictors associated with the response variable are considered to be in the neighborhood of a particular variable. After computing the neighborhoods for all the variables, an AND operator or an OR operator is used to determine the conditional independence of 2 traits i,j. (i.e., if *i *is in the neighborhood of *j *AND/OR *j *is in the neighborhood of *i*, they are conditionally dependent, given all other variables). The strength of the dependence can be measured by taking the maximum, minimum, or average of the 2 neighborhood measures between i,j. We performed LASSO regression for all the variables based on a cross-validated penalty parameter to estimate the sparse shrinkage coefficients.

## Results

### Data

Using the Gaussian graphical model for pedigrees and sparse graphical models for discrete and quantitative traits, we analyzed Genetic Analysis Workshop 18 (GAW18) data, which includes genome-wide association data for 400,000 SNPs, along with simulated and real phenotypic information SBP, DBP, hypertension, blood pressure medication use, and smoking status. The real data contained 939 individuals within 20 pedigrees at 4 time points. Missing data were present at all the time points. We excluded individuals with missing data for each of the time points and performed our analyses on the remaining data. For the analysis of data using the Gaussian graphical model for quantitative traits in pedigrees, we analyzed the first 3 time points for the 6 phenotypes in the real data. The fourth time point was excluded from the analysis because most of the data was missing for this time point.

For the sparse graphical model with discrete and continuous traits, we concentrated on chromosome 3. We used genome-wide association data for constructing the network. Two hundred replicates of simulated data for the 3 time points were available that were generated using the real pedigree structures. We used a single replicate of the simulated data for the phenotypes. Only the unrelated individuals from the first time point were used for this analysis. In the simulated data, a total of 1457 genetic variants were causal for either SBP or DBP across all the chromosomes. Of these 1457 causal variants, 188 variants were located on chromosome 3. We randomly sampled 20 of these 188 variants on chromosome 3 in our analysis.

### Gaussian graphical models for pedigrees

**Figure 1:**
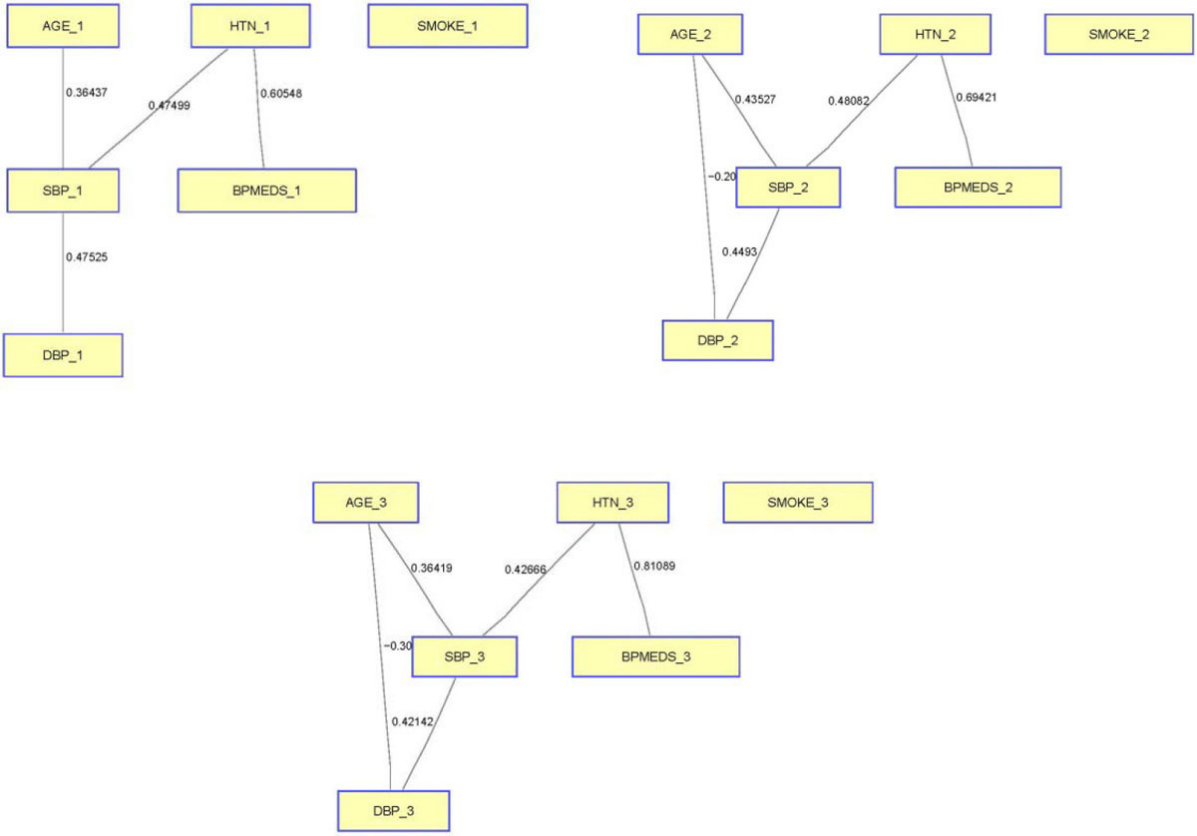
**Relationships among various traits accounting for correlation within and among pedigrees**. Shown are the relationships among the phenotypes in terms of a Gaussian graphical model. The edges correspond to the conditional relationship between 2 phenotypes, given all other phenotypes after accounting for the family structure. The 3 graphs represent sparse graphical models for each of the 3 time points.

We derived the graphical models of 6 traits and covariates, accounting for pedigree structure: age, SBP, DBP, hypertension, blood pressure medication use, and smoking status. Because hypertension, blood pressure medication use, and smoking status are discrete phenotypes, we transformed these variables into quantitative phenotypes using a logistic regression framework in which all the other phenotypes were regressed as dependent variables in the logistic model. At each time point, the graph shows the conditional relationships among the phenotypes. For example, in Figure 1, the graph for the second time point shows that age and DBP are conditionally negatively correlated given all the other phenotypes. The weight of the edge is the partial correlation between age and DBP, which was −0.2042. Similarly, the other edges point out the conditional relationships among the other phenotypes. The graph structure remained essentially the same for all 3 time points. Smoking status was not related to any of the other phenotypes at the 3 time points. Whereas DBP was inversely correlated with age, SBP was positively correlated with age.

### Sparse graphical models for binary and quantitative traits

**Figure 2:**
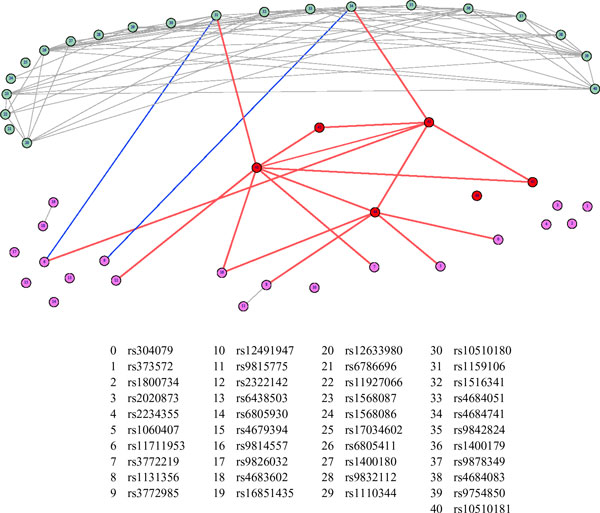
**Single-nucleotide polymorphism (SNP) network corresponding to 41 SNPs and 6 traits**. This figure shows the graphical model for 20 causal SNPs (pink, 0-19), 21 noncausal SNPs (green, 20-40), and 6 phenotypes (red, 41-46). The 6 phenotypes are age (41), hypertension (42), systolic blood pressure (SBP) (43), diastolic blood pressure (DBP) (44), blood pressure (BP) medication use (45), and smoking status (46). The red edges show the associations with the phenotypes. The blue edges show the linkage disequilibrium (LD) between the causal and noncausal variants. The gray edges show the LD among the noncausal variants.

We validated the sparse graphical methodology using the simulated genome-wide association data. Twenty of the 188 causal SNPs on chromosome 3 were randomly sampled. We also analyzed 21 consecutive noncausal SNPs from the same chromosome. The causal and noncausal SNPs analyzed are detailed in Figure 2. The graphical model also included the 6 phenotypes studied in the Gaussian model (age, SBP, DBP, hypertension, blood pressure medication use, and smoking status) for the first time point. Thus, our sparse graphical network model used 6 phenotypes, 20 causal SNPs, and 21 noncausal SNPs.

We performed LASSO regression using all 47 genetic and nongenetic factors and constructed the graph as described in the methods section. We used the AND operator for the conditional independence of 2 nodes to get a sparser graph. The strength of dependence was measured using the maximum measure of the 2 regression coefficients. Figure 2 shows the sparse graphical network of the phenotypes and the causal and noncausal SNPs. The phenotypes are coded in red, the causal SNPs in pink, and the noncausal SNPs in green. The 21 noncausal SNPs are in linkage disequilibrium (LD) with each other because of their proximity, which explains the huge number of edges between them. The network shows that the causal SNPs are linked with different phenotypes, but the noncausal SNPs are not linked to the phenotypes. However, 2 noncausal SNPs (rs1159106, rs4684741) were associated with the phenotypes. This can be explained by the noncausal SNPs being in low LD with 2 causal SNPs (rs11711953 and rs3772985, respectively), as shown by the blue edges in Figure 2. The $r^{2}$ values were 0.049 and 0.043, respectively. All of the phenotypes were interrelated, except for smoking status, which was independent of the other phenotypes and any genetic variants.

We also conducted additional validation of the proposed method where we randomly selected 21 noncausal SNPs from chromosome 3 that were not in LD with any of the causal SNPs or among themselves. All of the other phenotypes and the causal SNPs were as in the previous scenario. As expected, the resulting sparse graphical network (not shown) had no edges among the noncausal variants, and there were no edges connecting the causal variants and noncausal variants. The part of the network corresponding to the phenotypes and the causal SNPs was similar to the previous scenario.

## Discussion and conclusions

Graphical models provide an intuitive and straightforward way to visualize and use complex relationships among data. These models have mainly been used for analyzing case-control data among unrelated individuals. Here we have proposed a straightforward graphical method of accounting for correlation in pedigrees that can be used for decorrelating family data or, in general, for decorrelating correlated samples. If one is analyzing family data and needs to use a methodology that is suitable for case-control data with unrelated individuals, the data must first be decorrelated. In such cases, we can use the correlation structure identified from such graphical models as the variance-covariance matrix for the phenotypes. In this paper, we used c, induced correlation due to the shared environment, to be equal to 0.1. However, we have found that the method is robust to slight departures from the true value of c. The proposed model can be directly incorporated as a hierarchy into a Bayesian hierarchical model for simultaneously analyzing the phenotypes while taking into account the correlation among the family members.

We also explored a sparse network model that constructs an intuitive network graph including SNPs and discrete or continuous phenotypes. The network structure with the genetic and nongenetic factors is not perfect (in the sense that all the causal variants did not have links to the phenotypes). This may be because of the small sample size of the study. This raises an important question of whether we need to increase the number of pedigrees or the number of individuals within a pedigree. It is also important to note that one cannot assign a statistical significance (e.g., *p*-value) to the sparse graphical network as it is a data-driven structure. In the future, we intend to conduct follow-up studies investigating how to use the relationships between SNPs and between SNPs and phenotypes when analyzing genome-wide association data with multiple phenotypes.

## Competing interests

The authors declare that they have no competing interests.

## Authors' contributions

RT and SS conceived and designed the overall study, and RT conducted statistical analyses. RT and SS drafted the manuscript. Both authors read and approved the final manuscript.
